# Ovulation sources ROS to confer mutagenic activities on the *TP53* gene in the fallopian tube epithelium

**DOI:** 10.1016/j.neo.2024.101085

**Published:** 2024-12-04

**Authors:** Kanchana Subramani, Hsuan-Shun Huang, Pao-Chu Chen, Dah-Ching Ding, Tang-Yuan Chu

**Affiliations:** a Center for Prevention and Therapy of Gynecological Cancers, Department of Medical Research, Hualien Tzu Chi Hospital, Buddhist Tzu Chi Medical Foundation, Hualien, Taiwan, ROC; b Institute of Medical Sciences, Tzu Chi University, Hualien, Taiwan, ROC; c Department of Obstetrics & Gynecology, Hualien Tzu Chi Hospital, Buddhist Tzu Chi Medical Foundation, Hualien, Taiwan, ROC

**Keywords:** Fallopian tube fimbria, Follicular fluid, Reactive oxygen species, *TP53* mutation, Activation-induced cytidine deaminase, Mutagenesis

## Abstract

**Introduction:**

Epidemiological studies have implicated ovulation as a risk factor for ovarian high-grade serous carcinoma (HGSC) at the initiation stage. Precancerous lesions of HGSC commonly exhibit TP53 mutations attributed to DNA deamination and are frequently localized in the fallopian tube epithelium (FTE), a site regularly exposed to ovulatory follicular fluid (FF). This study aimed to assess the mutagenic potential of FF and investigate the expression levels and functional role of activation-induced cytidine deaminase (AID) following ovulation, along with the resulting TP53 DNA deamination.

**Methods:**

The mutagenic activity of FF toward premalignant and malignant FTE cells was determined using the hypoxanthine phosphoribosyl transferase (HPRT) mutation assay with or without AID knockdown. The sequential activation of AID, including expressional induction, nuclear localization, DNA binding, and deamination, was determined. AID inducers in FF were identified, and the times of action and signaling pathways were determined.

**Results:**

FF induced AID activation and *de novo* FTE cell mutagenesis in two waves of activity in accordance with post-ovulation FF exposure. The ERK-mediated early activity started at 2 min and peaked at 45 min, and the NF-κB-mediated late activity started at 6 h and peaked at 8.5 h after exposure. ROS, TNF-α, and estradiol, which are abundant in FF, all induced the two activities, while all activities were abolished by antioxidant cotreatment. AID physically bound to and biochemically deaminated the *TP53* gene, regardless of known mutational hotspots. It did not act on other prevalent tumor-suppressor genes of HGSC.

**Conclusion:**

This study revealed the ROS-dependent AID-mediated mutagenic activity of the ovulatory FF. The results filled up the missing link between ovulation and the initial *TP53* mutation and invited a strategy of antioxidation in prevention of HGSC.

## Graphical abstract

**Figure:**
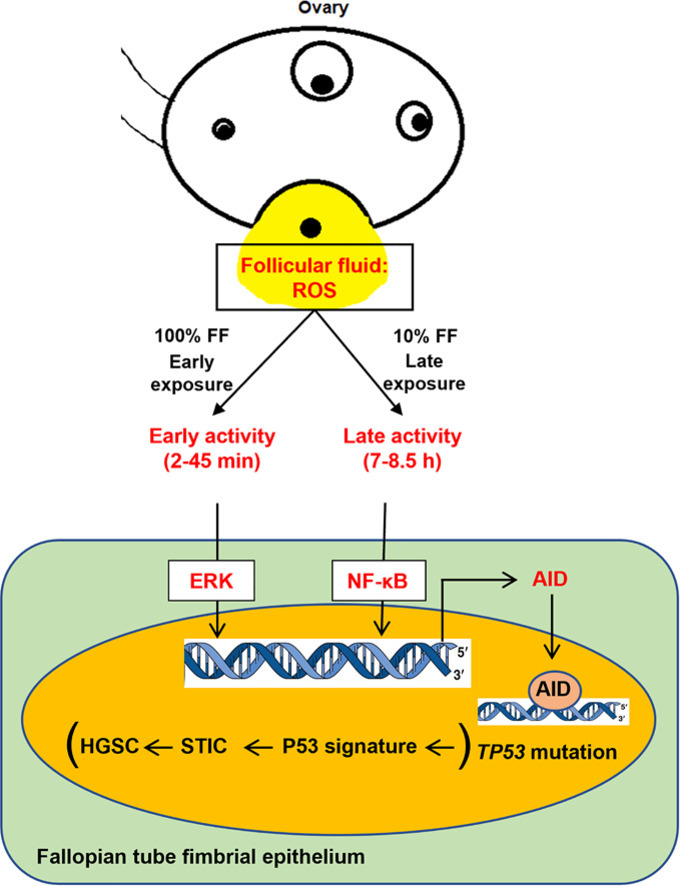

## Introduction

Epithelial ovarian cancer stands as the fifth leading cause of cancer-related death, with 207,252 deaths reported in 2018 worldwide, which represented approximately two-thirds of newly diagnosed cases [1,2]. High-grade serous carcinoma (HGSC) is the most prevalent and aggressive subtype, bearing the highest mortality rate, with the majority of cases going undetected until the late stage of disease [3]. The etiology and pathogenesis of HGSC, although still vague, have become clearer in two aspects: the primary origin at the distal fimbriated end of the fallopian tube [4,5], and incessant ovulation identified as the predominant risk factor [6,7].

The basis of incessant ovulation leading to HGSC has partly been uncovered [8]. During each ovulation and oocyte catchup, the fallopian tube fimbrial epithelium (FTE) is exposed to the ovulatory follicular fluid (FF), which contains high concentrations of oncogenic factors, including reactive oxygen species (ROS), inflammatory cytokines, and growth factors [9,10]. Thus, the FF-exposed FTE experiences oxidative stress, leading to DNA double-strand breaks [11,12]. Growth factors, such as IGF2 and HGF, in the FF further induce the progressive transformation of the p53/Rb-initiated FTE cells in a serial development of stemness activation, clonal expansion, anchorage-independent growth, and tumorigenesis [[13], [14], [15]]. One major missing piece of information is the initiation of FTE cell transformation, especially the cause and mechanism of initial genetic mutations.

In 96 % of HGSC cases [16] and in the majority of precancerous lesions in FTE, such as the “p53 signature” and serous tubal intraepithelial carcinoma, [[17], [18], [19]] there is a pathological mutation in the *TP53* gene. Among them, 62 % are missense mutations and 35 % are non-missense mutations. These mutations result in loss- or gain-of-function with dominant negative effects of the p53 protein [20]. The first identifiable precancerous lesion in FTE, “p53 signature”, typically carries gain-of-function *TP53* mutations resulting in a stabilized p53 accumulated in the nucleus, [18] presumably as a result of clonal expansion of a mutant stem or progenitor cell. In the analysis of 293 whole-exome datasets of HGSC tumors, a total of 33,859 somatic mutations were identified. Among these, 32.5 % were characterized as C:G to T:A transitions, [21] which represent the most common mutational outcome associated with activation-induced cytidine deaminase (AID) function [22].

AID is a DNA editing and natural mutator enzyme encoded by the *AICDA* gene. Physiologically, AID triggers somatic hypermutation and class switch recombination within the immunoglobulin (Ig) genes of B lymphocytes [23]. Recent studies have revealed that the genotoxic effects mediated by AID extend beyond Ig genes to target a diverse range of other genes. Due to its involvement in point mutations and chromosomal rearrangements, AID is recognized as a key mutator enzyme implicated in various cancer types, including hepatocellular carcinoma, cholangiocarcinoma, gastric cancer, colitis-associated colorectal cancer, and chronic lymphocytic leukemia [24,25]. There is suggestive evidence that AID might serve as the missing link between inflammation induced by ovulation, DNA mutation, and carcinogenesis of the fallopian tube epithelium [21]. Nevertheless, the mechanism by which ovulation induces AID activation, whether AID is accountable for the mutagenesis of FTE, and whether *TP53* is targeted remain unknown. We propose that the exposure of the FTE to mutagens present in ovulatory FF could potentially induce *de novo* mutagenesis of the *TP53* gene by activating AID.

In this study, we used a hypoxanthine-guanine phospho-ribosyltransferase (HPRT) gene mutation assay to assess the mutagenic activity of the FF. Furthermore, we explored FF-induced AID activation in the FTE during early and late exposure to FF after ovulation. Furthermore, the underlying pathways of AID activation, binding of AID to the *TP53* gene, and subsequent deamination mutations were determined.

## Methods

### Cell culture

The cell lines used in this study included premalignant human fimbrial epithelial cell lines (FE25, FT282-V, and FT282-CCNE1), a transformed Chinese hamster ovary cell line (CHO-K1), and a human ovarian cancer cell line (A2780). The FE25 cell line was established in our laboratory through the transduction of HPV (*E6*/*E7*) and *hTERT* genes and was non-transformed at the early passage (P20) and transformed after late passage (P90) [26]. The FT282-CCNE1 cell line was established by transducing the *CCNE1* gene to a p53-R175H/hTERT-immortalized FTE cell FT282, and FT282-V served as the vector control [27]. The three FTE cell lines were cultured in a medium containing a 1:1 ratio of MCDB/M199 (Sigma) supplemented with 10 % FBS as well as 100 IU/ml penicillin and 100 μg/ml streptomycin. The CHO-K1 cell line was obtained from ATCC and maintained in F-12K medium (Gibco-Thermo Fisher Scientific) supplemented with 10 % FBS. The A2780 cell line, an epithelial ovarian cancer cell line expressing the wild-type *TP53* gene, was cultured in RPMI 1640 medium (11875119; Gibco-Thermo Fisher Scientific) supplemented with 10 % FBS and 1 % P/S.

### Clinical specimens

Approval for the collection of clinical specimens was obtained from the Institutional Review Board of Tzu Chi General Hospital, Hualien, Taiwan (TCGH-IRB #93-025). Each donor provided informed consent before participation. FF aspirates were obtained from women undergoing oocyte retrieval and *in vitro* fertilization, as described previously [11]. The fallopian tube specimens utilized in this study were collected from patients undergoing salpingectomy for benign conditions, as previously described [11].

### HPRT gene mutation assay

The HPRT gene mutation assay was conducted following the method originally described by George E. Johnson, [28] with slight modifications, as outlined below:

(1) Mutant cleansing: The cells were cultured in media supplemented with hypoxanthine aminopterin thymidine (H0262; Sigma) and hypoxanthine thymidine (H0137; Sigma) for 3–4 days to inhibit the cells from relying on the endogenous pathway for nucleotide synthesis. This allows for the survival of cells capable of nucleotide synthesis solely through HPRT. Additionally, cells with pre-existing HPRT mutations were eliminated during this process.
(2) Treatment with mutagens: The pre-cleaned cells were exposed to different treatments, including ethyl methane sulfonate (EMS) (M0880; Sigma) and FF for 24 h. After treatment, the cells were subcultured for seven passages once they reached confluency.
(3) HPRT mutant selection and colony formation: Following treatment, cells were subjected to HPRT mutant selection and colony formation assays. The cells were cultured in media containing or lacking 60 μM of 6-thioguanine (6-TG) (A4882; Sigma) for the FTE cells and 10 μM for CHO-K1. Normal HPRT gene-containing cells incorporated 6-TG and succumbed to toxicity, whereas HPRT mutant cells exhibited resistance to 6-TG and formed colonies.
(4) Scoring method: HPRT mutant colonies were fixed with 4 % paraformaldehyde for 10 min, stained with 0.5 % crystal violet, and viable colonies larger than 50 μm were scored.
(5) Calculation of mutation frequency: The mutation frequency was determined based on the number of HPRT mutant colonies corrected for plating efficiency. A cell culture dish without 6-TG was used to calculate the plating efficiency of the cells, and a dish with 6-TG was used to calculate the mutation frequency.

### Superovulation of mice

Wild-type C57BL/6J mice were obtained from the Taiwan National Laboratory Animal Center. Progesterone receptor gene (Pgr)-knockout C57BL/6JNarl-Pgrtm1Tyc/Tmc mice were generated in our laboratory using CRISPR/Cas9 technology. This strain was created by the targeted deletion of the 3′ half sequences of the *Pgr1* gene. For the induction of superovulation, mice were intraperitoneally injected with 5 IU of pregnant mare serum gonadotropin (PMSG) followed by 5 IU of human chorionic gonadotropin (hCG) in 100 μl of PBS, as previously described [29].

### Culture of human fimbria

To culture human fimbria tissue *ex vivo*, the fallopian tube was opened longitudinally along the lumen. The fimbria were then dissected into 1 × 1 cm pieces and incubated in RPMI 1640 medium with or without FF or candidate factor ± specific inhibitors.

### Cytokine and inhibitor treatment

The FE25 cells were cultured with or without recombinant cytokines, including TNF-α, IL-1β, TGF β2, IL-6, IL-8, IP-10 (Peprotech), and Estradiol (E1024; Sigma). Additionally, FE25 cells were cultured with or without pretreatment with SN50 (an NF-κB inhibitor, 481480; Sigma) and ulixertinib (an ERK inhibitor, BVD523, MedChemExpress) for 2 h.

### RNA extraction and quantitative real-time PCR (qRT-PCR)

RNA was extracted using the GeneJET RNA kit (K0731; Thermo Fisher Scientific). cDNA was synthesized using a Thermo Scientific RevertAid H Minus cDNA Synthesis Kit (K1631; Thermo Fisher Scientific). The qRT-PCR was conducted using the fast SYBR® Premix EX Taqᵀᴹ (RR420L; Takara), and the analysis utilized the following PCR primer pairs (see Suppl. Table 2): AID Forward: 5′GGGAACCCCAACCTCTCTCT, AID Reverse: 5′CCTTGCGGTCCTCACAGAAG. The qRT-PCR procedure involved an initial denaturation step at 95°C for 5 min, followed by 40–45 cycles of denaturation at 95°C for 20 s, annealing at 55°C for 20 s, and extension at 72°C for 1 min. The fold change was determined using the 2^–∆∆Ct^ method based on the Ct values obtained from the qRT-PCR analysis.

### Immunohistochemistry, immunofluorescence, and western blot

The paraffin tissue sections, with a thickness of 4 μm, were subjected to deparaffinization and subsequently stained. For immunohistochemistry (IHC) and immunofluorescence analyses, the antibodies were AID (ZA001; Thermo Fisher Scientific, 1:50) and Anti-NF-κB p65 (ab32536; Abcam, 1:200). For western blot analysis, following SDS-PAGE and nitrocellulose membrane transfer, AID (ZA001; Thermo Fisher Scientific, 4:1000), Anti-NF-κB p65 (ab32536; Abcam, 1:200), and Phospho-p44/42 (9101; Cell Signaling 1:1,000) antibodies were used.

### Knockdown using shRNA lentiviral vectors

AID in FE25 cells was knocked down using lentivirus-mediated shRNA. Target-specific shAID1 (sequence: GATGACTTACGAGACGCATTT), shAID2 (sequence: GCCATCATGACCTTCAAAGAT), and control shLuc (sequence: CAAATCACAGAATCGTCGTAT) were purchased from the National RNAi Core Facility (Academia, Sinica, Taiwan).

### Chromatin immunoprecipitation (ChIP) assay

A ChIP assay was conducted on FE25 cells following treatment with FF using a ChIP assay kit (17-295; Sigma-Aldrich). Upon reaching the required time points, the cells were fixed with 37 % formaldehyde for 10 min to establish DNA-histone crosslinks. Subsequently, the cells were washed twice with PBS containing a protease inhibitor cocktail (R1321; Fermentas). The cells were then scraped, and an SDS lysis buffer with a protease inhibitory cocktail was added. After incubation on ice for 10 min, the cells were sonicated with four 10-s pulses. The sonicated cells were centrifuged at 10,000 rcf (g) for 5 min. A 20 μl sample was taken from the supernatant for positive control DNA isolation, which involved reverse crosslinking with 1 μl of 5 M NaCl. The remaining supernatant was subjected to immunoprecipitation by adding an AID antibody (ZA001; Thermo Fisher Scientific, 2 μg) or an anti-mouse IgG antibody (7076; Cell Signaling, 1:10,000), followed by overnight incubation at 4°C with rotation. After incubation, protein A agarose beads were added and incubated for 1 h at 4°C. The agarose beads containing the AID-DNA complex were washed with the appropriate buffers, and the complex was eluted from the beads using an elution buffer. Following elution, the complex was reverse-crosslinked with 5 M NaCl for 4 h, and DNA was isolated using a Genomic DNA isolation kit (NA026-0100; GeneDireX). Subsequently, the DNA was subjected to PCR targeting *TP53* and other tumor suppressors, as shown in Suppl. Table 2.

### Uracil-qRT-PCR

Uracil-qRT-PCR was performed according to a previously described protocol [30,31]. Briefly, 50 ng of genomic DNA template was added to the PCR mixture. Either PfuTurbo Hotstart (600320; Agilent Technologies) or PfuTurbo Cx Hotstart DNA polymerase (600410; Agilent Technologies) was used. PfuTurbo Cx Hotstart DNA polymerase was specifically designed with a point mutation to overcome uracil stalling during PCR amplification, enabling the polymerase to read uracil on the template strand. qRT-PCR was performed using a QuantStudio 6 Flex system (Thermo Fisher Scientific). The primers used are outlined in Suppl. Table 2. For data analysis, Ct values obtained with PfuTurbo Hotstart DNA polymerase were used as a reference, and the 2^-ΔΔCt^ method was employed to determine the relative uracil level.

### Statistical analysis

Statistical analyses were performed using GraphPad Prism, Microsoft Excel 2010, and ImageJ software. For statistical comparisons, the data were analyzed using two-tailed paired and unpaired Student's t-tests. Differences were considered statistically significant at p < 0.05.

## Results

**Fig. 1:**
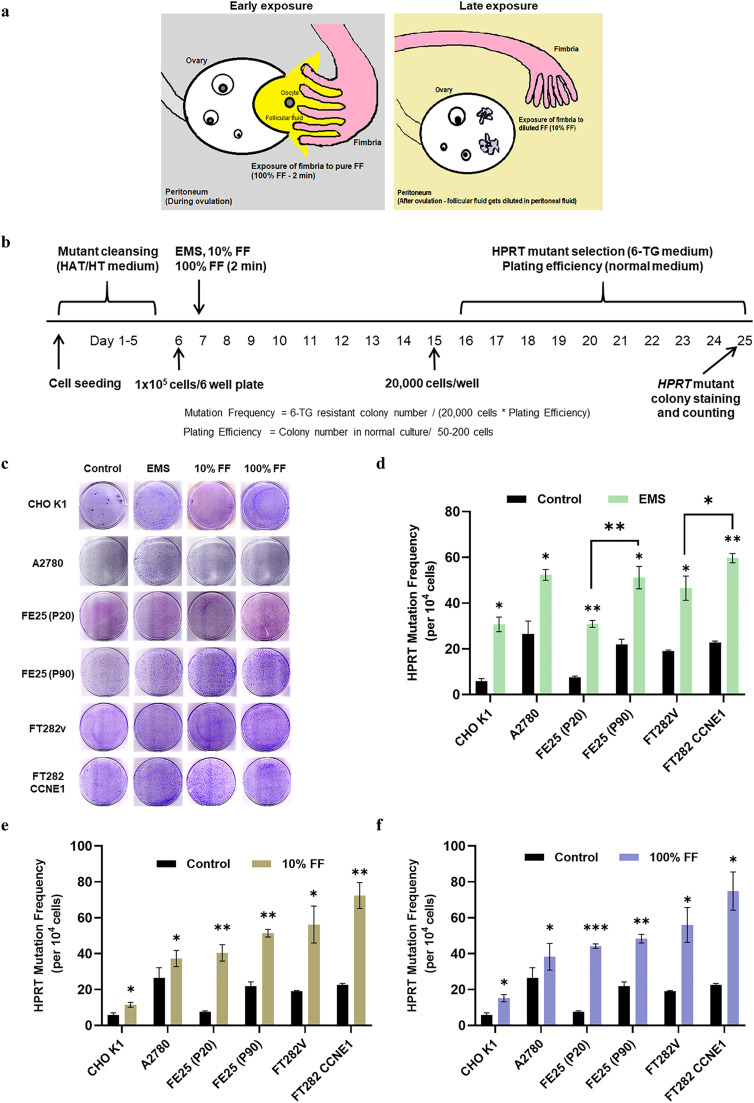
Ovulatory FF is mutagenic to FTE cells: Less transformed cells are more vulnerable. (a) Physiological scenarios of FF exposure used in the study, with two concentrations of FF as 100 % and 10 % termed Early and Late exposure, respectively. (b) Timeline of HPRT mutation assay. The HAT/HT-supplemented, *HPRT* mutant-pre-cleansed cells were treated with EMS (500 μM), 10 % FF (no wash), and 100 % FF (wash after 2 min) for 24 h. The cells were then washed and passaged for 7 days upon reaching confluency. Later, the cells were seeded in the 6-TG medium for HPRT mutant selection and in normal medium for plating efficiency. The colonies formed were stained and counted. The fraction of 6-TG-resistant colonies and the number of cells seeded corrected for plating efficiency yielded the mutation frequency. (c) Representative images of HPRT colonies formed in different cell lines with individual treatment groups. (d, e, f) The graphical representation of calculated *HPRT* gene mutation frequency in different cell lines with EMS (C), 10 % FF (D), and 100 % FF (E) treatment compared to control. * p < 0.05, ** p < 0.01, *** p < 0.001 by two-tailed paired Student's t-test. HAT (Hypoxanthine aminopterin thymidine), HT (Hypoxanthine thymidine).

1) **Ovulatory FF is mutagenic to FTE cells: Less transformed cells are more vulnerable**
  The mutagenic activity of FF was determined in different cell lines using an HPRT gene mutation assay. To imitate the physiological state of FF release after ovulation and subsequent dilution into the peritoneal fluid, we designed two sets of exposure: immediate, short-duration (2 min) exposure with pure FF (designated as Early exposure) and long-lasting (no wash) exposure with 10 % diluted FF (designated as Late exposure) (Fig. 1a). Four FTE cell lines in different states of transformation, one epithelial ovarian cancer cell line, and one CHO-K1 control cell line were tested. As shown in Fig. 1b, after removing the pre-existing HPRT mutants, the cells were subjected to Early exposure, Late exposure, or EMS mutagen control. HPRT *de novo*-mutated colonies resistant to 6-TG were selected (Fig. 1c), and the mutation frequencies in terms of mutants per 10^4^ cells were determined after adjusting the plating efficiencies (Table 1 and Suppl. Table 1). We found that transformed FTE cells, either through late cell passages (FE25-P90)(26) or by introducing the *CCNE1* oncogene (FT282-CCNE1) [27], were more vulnerable to the standard EMS mutagen compared to their non-transformed counterparts, FE25-P20 and FT282-V (51.1 ± 4.8 vs. 30.9 ± 1.5 colonies, and 59.6 ± 2.0 vs. 46.5 ± 5.3 colonies, respectively; p = 0.0024 and 0.0161; Fig. 1d). The same was true for mutagenesis induced by Early and Late FF exposure (Fig. 1e and 1f). More importantly, compared to no exposure, Early and Late FF exposure markedly increased the mutation frequency of non-transformed FE25 cells by 5.8- and 5.3-fold, respectively (Table 1). Other transformed or non-transformed FTE cells also showed multiple-fold increases, whereas malignant epithelial ovarian cancer cells (A2780) only showed a modest increase (Fig. 1e and 1f). These results indicate that exposure to ovulatory FF in either the Early or Late exposure scenarios can induce a remarkable increase in *de novo* mutations in FTE cells, particularly in cells in a less transformed state.

**Table 1:** The HPRT mutation frequency of individual treatment groups in different cell lines.

Treatment groups	Concentration	HPRT Mutation Frequency per 10^4^ cells (Fold change)					
		CHO K1	A2780	FE25 (P20)	FE25 (P90)	FT282V	FT282 CCNE1
**Control**		5.8 (1.0)	26.5 (1.0)	7.5 (1.0)	21.9 (1.0)	19.0 (1.0)	22.7 (1.0)
**EMS**	500 µM	30.7 (5.4)	52.3 (2.0)	30.9 (4.0)	51.1 (2.3)	46.5 (2.3)	59.6 (2.5)
**FF**	10 %	11.5 (2.0)	37.3 (1.4)	40.4 (5.3)	51.4 (2.3)	56.2 (2.9)	72.4 (3.1)
**FF**	100 %	15.2 (2.6)	38.3 (1.4)	44.2 (5.8)	48.4 (2.1)	56.0 (2.9)	74.8 (3.2)

**Fig. 2:**
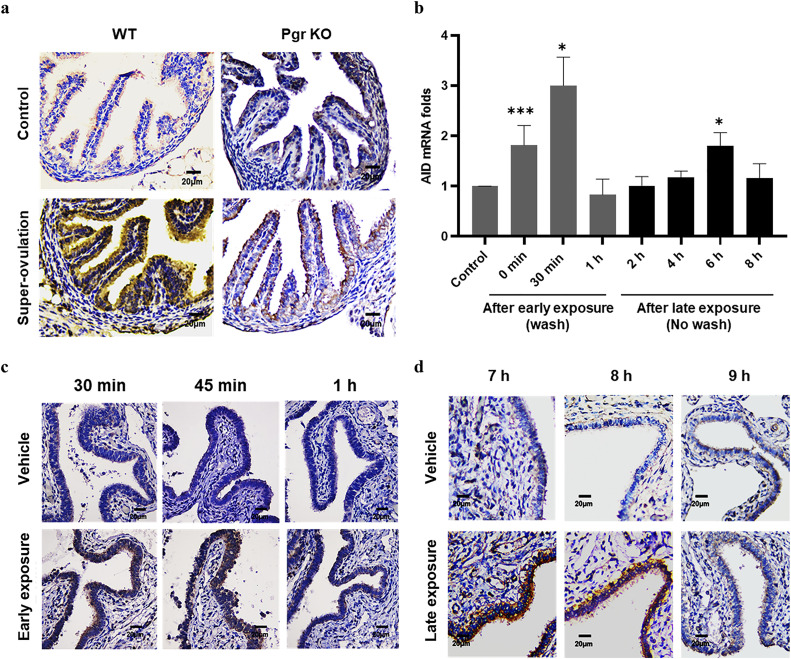
Ovulatory FF induces two waves of AID expression in the FTE in accordance with Early and Late exposure after ovulation. (a) IHC staining of AID in oviduct sections of wild-type (WT) and progesterone knockout (*Pgr* KO) mice with or without superovulation. (b) The AID mRNA expression of FE25 cells treated with 100 % FF (2 min, washed) checked at different points from 0 min to 1 h and with 10 % FF (no wash) checked from 2–8 h. IHC staining of AID in human fallopian tube fimbria *ex vivo* after exposure to vehicle, 100 % FF (2 min, washed) checked at different time intervals from 30 min to 1 h (c), and 10 % FF (no wash) checked from 7–9 h (d). * p < 0.05, ** p < 0.01, *** p < 0.001 by two-tailed paired Student's t-test.
  In non-transformed human FE25 cells, two waves of increase of AID mRNA were observed after Early or Late FF exposure: one peaked 30 min after Early exposure and the other peaked 6 h after Late exposure (Fig. 2b). FF exposure to *ex vivo*-cultured human fallopian tube fimbria resulted in two similar waves of upregulation, 30–45 min after Early exposure (Fig. 2c) and 7–8 h after Late exposure (Fig. 2d). Thus, exposure of the fallopian tube fimbria to ovulatory FF leads to the upregulation of AID in accordance with the physiological time sequence of ovulation.
3) **ROS in FF is responsible for AID induction activity**
  Previously, we discovered that half of human FF samples carried high levels of ROS, and in contrast to ROS-low FF, ROS-high FF conferred transformation of FTE cells *in vitro* and *in vivo* [11]. We wondered whether ROS in FF are responsible for AID induction and mutagenesis activity. Indeed, H_2_O_2_, like FF, induced the same AID mRNA and protein expression in FE25 cells and fimbria tissues in the two modes of exposure, and treatment with N-acetyl-L-cysteine (NAC) antioxidant abolished the increased expression levels *in vitro* and *ex vivo* (Fig. 3a and 3b).

EMS (Ethyl methane sulfonate), FF (Follicular fluid)
2) **Ovulatory FF induces two waves of AID expression in the FTE in accordance with Early and Late exposure after ovulation**
  We investigated whether FF-induced mutagenic activity was mediated by AID. As shown in Fig. 2a, an obvious increase in FTE AID expression was observed when the mice were subjected to PMSG/hCG superovulation. In contrast, superovulation in progesterone receptor gene knockout mice, in which mature follicles did not rupture, did not result in increased AID expression. Thus, excessive ovulation can upregulate AID expression in FTE upon exposure to the FF released during ovulation.

**Fig. 3:**
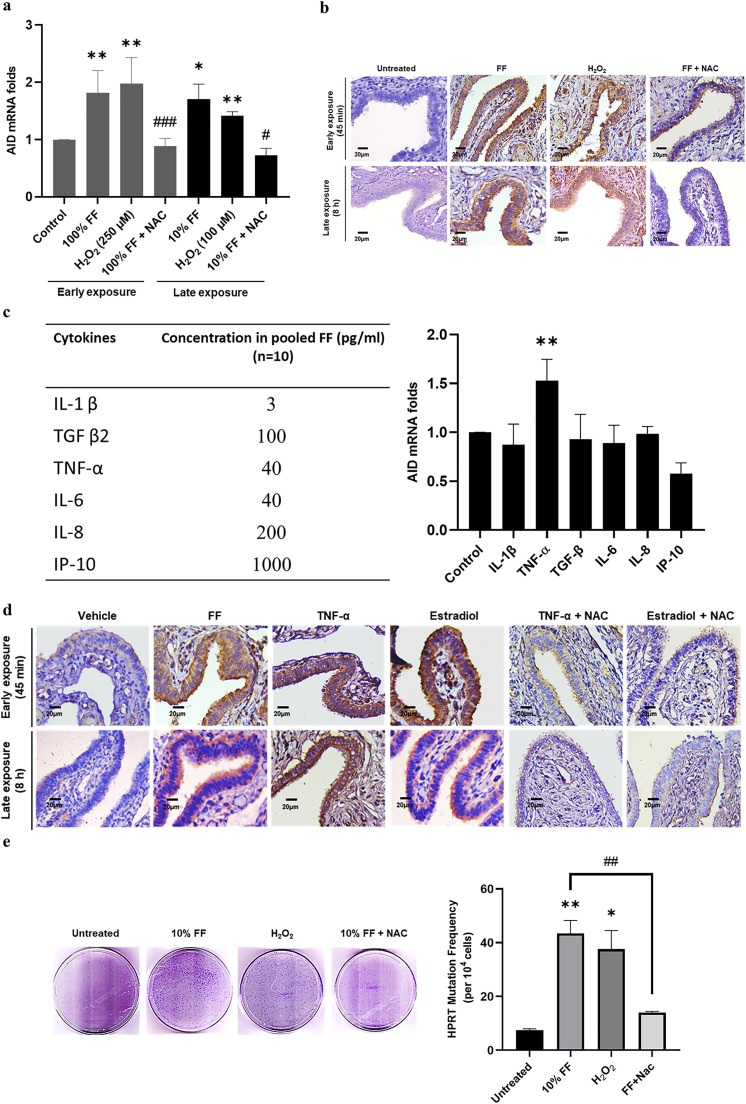
ROS in FF are indispensable for AID induction and mutagenesis activity. (a) AID mRNA expression in FE25 cells exposed to the following (from left to right, after Control): 100 % FF, H_2_O_2_ (250 μM), and 100 % FF with 500 μM N-acetyl-L-cysteine (NAC; an antioxidant) cotreatment for 2 min (dark grey bars); 10 % FF, H_2_O_2_ (100 μM), and 10 % FF with NAC cotreatment for 6 h (black bars). (b & d) Upper panels: Exposure to 100 % FF, H_2_O_2_ (250 µM), TNF-α (400 pg/ml), and estradiol (100 nM) with or without NAC cotreatment for 2 min, followed by washing and checking after 45 min. Lower panels: Exposure to 10 % FF, H_2_O_2_ (100 µM), TNF-α (40 pg/ml), and estradiol (10 nM) with or without NAC cotreatment for 8 h. (c) Concentration of cytokines in FF (left). AID mRNA expression in FE25 cells exposed to different cytokines for 6 h, associated with their concentration in FF (right). (e) Representative images (left) and graphical representation (right) of HPRT colonies and mutation frequency in FE25 cells exposed to 10 % FF with or without NAC (500 μM) cotreatment and H_2_O_2_ (100 μM). * p < 0.05, ** p < 0.01, *** p < 0.001 compared to control; ^#^ p < 0.05, ^# #^ p < 0.01, ^# # #^ p < 0.001 comparison of 100 % FF vs. 100 % FF + NAC and 10 % FF vs. 10 % FF + NAC by two-tailed paired Student's t-test.
4) **TNF-α and estrogen in FF also induce AID expression in a ROS-dependent manner**
  Reportedly, AID expression can be induced by cytokines and estrogen [32,33,34], both of which are abundant in FF [9]. Using a cytokine array, we found that six cytokines (TNF-α, IL-1β, TGF β2, IL-6, IL-8, and IP-10) were present at high levels in a pooled FF sample from 10 women (Fig. 3c, left). In concentrations equivalent to the levels in 10 % FF, as shown in Fig. 3c, only TNF-α promoted AID mRNA expression in FE25 cells. Meanwhile, owing to the loss of estrogen receptor, FE25 cells failed to respond to estradiol to induce AID (data not shown). However, estradiol, TNF-α, and Early or Late exposure to FF readily induced AID expression in human fimbria tissue. Interestingly, NAC cotreatment abolished all these effects (Fig. 3d). Thus, ROS are directly or indirectly responsible for the upregulation of AID upon exposure to ovulatory FF.
5) **ROS are indispensable for the mutagenesis activity mediated by AID**
  Given the critical role of ROS, we tested whether antioxidants could diminish the mutagenic activity of FF. As expected, NAC significantly compromised the mutagenic activity conferred by 10 % FF and 100 µM H_2_O_2_ (a concentration equivalent to the ROS levels in 10 % FF) [35] (Fig. 3e). Thus, the FF-ROS signal confers both AID upregulation and subsequent mutagenesis in FTE cells after ovulatory exposure.
  We further explored the role of AID in ROS-mediated FF-induced mutagenesis using shRNA knockdown. Two AID knockdown FE25 cell clones (Fig. 4a) subjected to 10 % FF or 100 µM H_2_O_2_ exposure were tested for HPRT mutagenesis. As shown in Fig. 4b and 4c, the capacity to induce *de novo* HPRT mutations by either FF or H_2_O_2_ was compromised by AID knockdown.

**Fig. 4:**
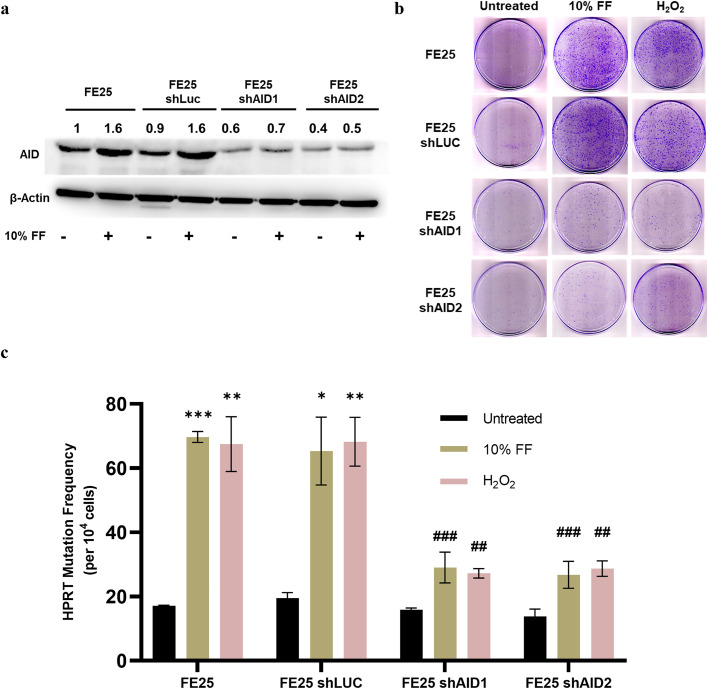
AID is required for mutagenesis by FF-ROS. (a) Confirmation of AID knockdown in FE25 cells by western blot analysis. (b & c) Representative images and graphical representation of HPRT colonies and mutation frequency in FE25 cells with or without AID knockdown exposed to 10 % FF and H_2_O_2_ (100 μM). * p < 0.05, ** p < 0.01, *** p < 0.001 compared to the same untreated cells; ^#^ p < 0.05, ^# #^ p < 0.01, ^# # #^ p < 0.001 comparison of treatment groups in FE25 cells and FE25 shAID1 & FE25 shAID2 cells by two-tailed paired Student's t-test.
6) **The FF-ROS mutagenic activity of Late exposure is mediated by NF-κB, and that of Early exposure is mediated by ERK**
  To elucidate the signal pathway through which FF-ROS induces two waves of AID expression, we first focused on the known NF-κB pathway of AID activation [36]. NF-κB activation was evident in the Late-exposure cells, indicated by its nuclear presence and elevated levels of phosphorylated proteins (Fig. 5b). Interestingly, pretreatment with the NF-κB inhibitor SN50 abolished the AID induction by the Late exposure of FF and H_2_O_2_, but not by the Early exposure (Fig. 5a). The signaling pathways for Early exposure were further investigated to determine the immediate-early response pathway EARK/MAPK [37]. Indeed, pretreatment with the ERK1/2 inhibitor (ulixertinib) abolished AID mRNA induction by Early but not Late exposure (Fig. 5a). An increase in p-ERK1/2 levels was evident after 1 min of 100 % FF exposure prior to the onset of AID expression (Fig. 5c). The findings suggest that AID induction by Late FF exposure follows the NF-κB pathway, whereas Early exposure induces AID expression *via* the ERK pathway.

**Fig. 5:**
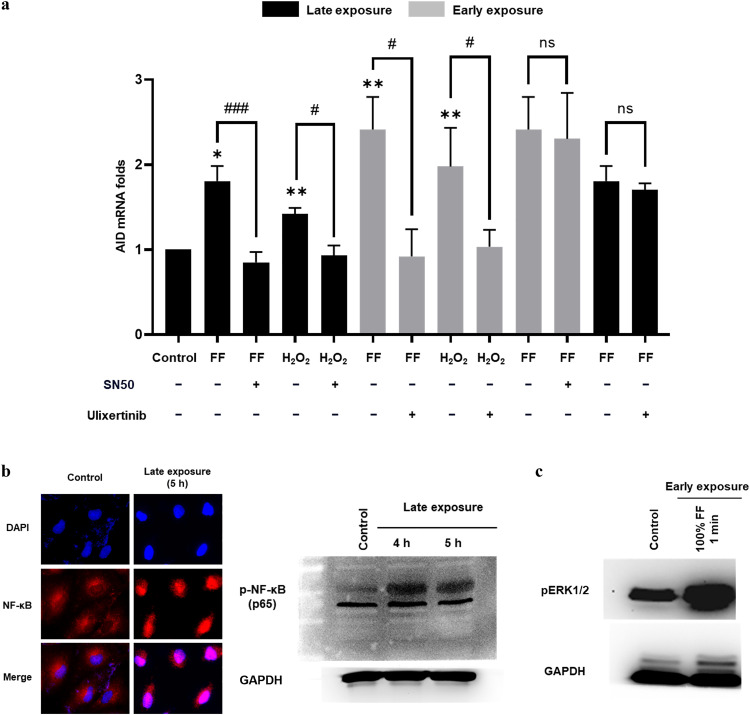
The FF-ROS mutagenic activity of Late exposure is mediated by NF-κB, and that of Early exposure is mediated by ERK. (a) The AID mRNA expression in FE25 cells treated with 10 % FF, H_2_O_2_ (100 μM) for 6 h, 100 % FF, and H_2_O_2_ (250 μM) for 2 min with and without SN50 (NF-κB inhibitor) or ulixertinib (ERK1/2 inhibitor) at a concentration of 100 nM with 2 h pretreatment. (b) Immunofluorescence staining of NF-κB in FE25 cells with or without 10 % FF treatment for 5 h. (c) Western blot analysis of phosphorylated NF-κB with or without 10 % FF treatment. (d) Western blot analysis of phosphorylated ERK with or without 100 % FF for 1 min in FE25 cells. * p < 0.05, ** p < 0.01, *** p < 0.001 compared to control; ^#^ p < 0.05, ^# #^ p < 0.01, ^# # #^ p < 0.001 comparisons of FF and H_2_O_2_ with or without inhibitors by two-tailed paired Student's t-test.
7) **FF exposure activates AID to bind to and deaminate the *TP53* gene but not the other tumor suppressor genes of HGSC**
  We further investigated the nucleus entry and DNA targeting of AID after FF exposure. Following the Late exposure, the AID protein appeared in the cytoplasm after 7 h, translocated to the nucleus by 8 h, peaked at 8.5 h, and diminished 30 min thereafter. Similarly, following Early exposure, AID translocated to the nucleus within 45 min and disappeared within 15 min (Fig. 6a).

**Fig. 6:**
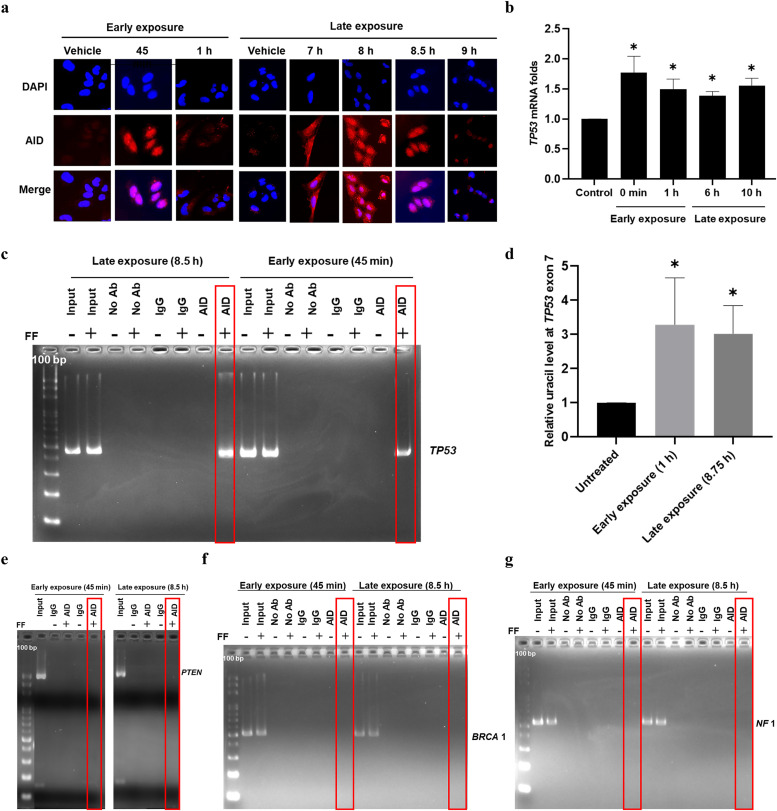
FF exposure activates AID to bind to and deaminate the *TP53* gene but not other tumor suppressor genes of HGSC. (a) Immunofluorescence staining of AID in FE25 cells with or without 100 % FF (2 min, washed) checked after 45 min to 1 h (left) and 10 % FF (no wash) from 7–9 h (right) to identify the time of AID nuclear localization. Scale: 20 µm. (b) *TP53* mRNA expression in FE25 cells treated with 100 % FF and 10 % FF to identify the time of *TP53* active transcription. (c, e, f, g) ChIP-PCR in FE25 cells at AID nuclear localization time points with or without 100 % FF and 10 % FF treatment. Chromatin was immunoprecipitated with AID and IgG (control) antibodies, and PCR was used to detect AID-bound DNA, targeting the *TP53* (c), *PTEN* (e), *BRCA*1 (f), and *NF*1 (g) genes. The red boxes with bands indicate AID binding to specific genes as positive, and the ones without bands indicate AID binding to specific genes as negative. (d) The relative level of uracil at *TP53* exon 7 measured by uracil-qRT-PCR to check the deamination status after AID binding in FE25 cells treated with or without 100 % FF (left) and 10 % FF (right). * p < 0.05, ** p < 0.01, *** p < 0.001 by two-tailed paired Student's t-test.
  AID preferentially binds to single-stranded DNA [38]. Actively transcribing genes involve the unwinding of the DNA double helix in the transcription bubble and typically possess an "open" chromatin structure that facilitates the binding of trans-activating factors. We found that *TP53* mRNA levels increased after FF exposure at the exact time corresponding to the nuclear localization of AID (Fig. 6b). Using ChIP-PCR, we revealed the binding of AID to *TP53* DNA after either Early or Late exposure, at the exact time of AID nuclear residence (Fig. 6c). Deamination of cytosine to uracil in *TP53* gene was quantitated by uracil-qRT-PCR. After Early or Late FF exposure, an increase in uracil levels was observed at the tested bound sequences at exon 7 (Fig. 6d). Thus, *TP53*-bound AID is enzymatically active, resulting in cytosine-to-uracil mutations.
  Moreover, we investigated whether AID binds to other tumor suppressor genes implicated in HGSC, such as *PTEN, NF1, BRCA1, BRCA2*, and *CDK12* [16]. We assessed the transcriptional activity of these genes following the two modes of FF exposure in FE25 cells. None of the genes responded to the FF exposures (Fig. S1a); except that Late FF exposure led to an increase in *PTEN* expression, aligning with the timeframe of AID nuclear localization (Fig. S1b). However, ChIP-PCR analysis failed to reveal the binding of AID to either *PTEN* or the two frequently mutated genes, *BRCA1* and *NF1* (Fig. 6e–g). Collectively, these results suggest that FF-induced AID acts with *TP53* but not with other tumor suppressor genes in HGSC. We were also curious whether the AID binding sites coincided with the mutation hot spots of the *TP53* gene [20]. As depicted in Fig. 7a and 7b, FF exposure induced the binding of AID to the *TP53* gene in both the mutation-hot (exons 5, 6, and 7) and mutation-cold (exons 1, 2, and 11) exons.

**Fig. 7:**
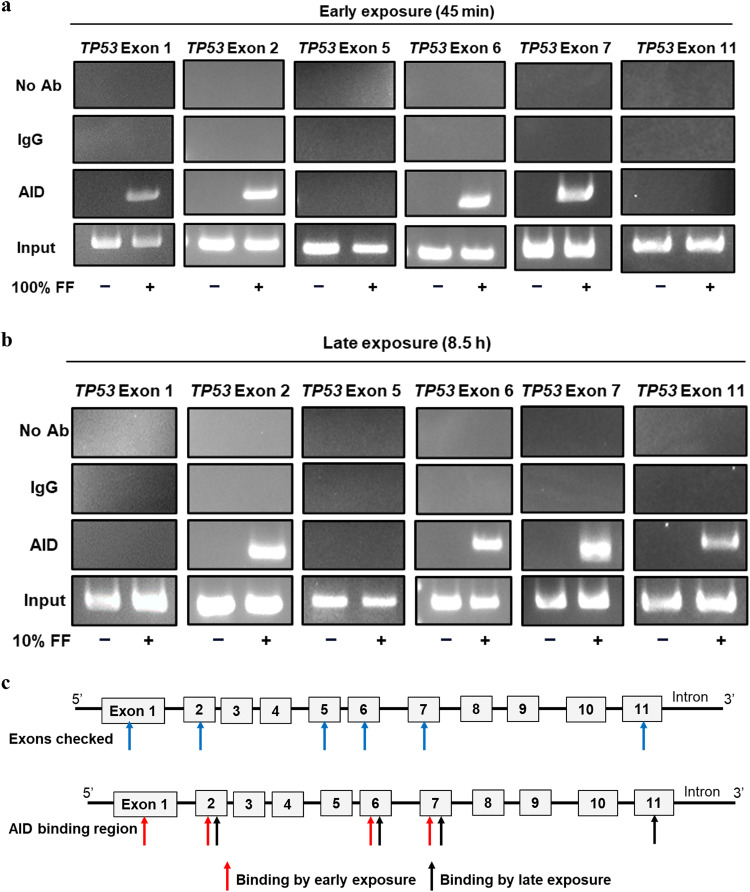
The two waves of FF-induced AID binding to the *TP53* gene, irrespective of the mutational hotspots. (a & b) ChIP-PCR in FE25 cells at AID nuclear localization time points with or without 100 % FF (a) and 10 % FF (b) treatment. Chromatin was immunoprecipitated with AID and IgG (control) antibodies, and PCR was used to detect AID-bound DNA, targeting the *TP53* gene at various exonic regions, including mutational hotspots (exon 5, 6, and 7) and non-hotspots (exon 1, 2, and 11). (c) Map of 100 % and 10 % FF-induced AID binding to *TP53* exons.

## Discussion

This study determined the mutagenic activity of human follicular fluid (FF). Surprisingly, the mutagenic potential of FF was found to be as high as 4.42 mutations in 1000 cells per exposure, surpassing the standard EMS mutagen at 500 μM (Table 1). This represents the first documented instance of endogenous mutagenic activity in tissue fluids to our knowledge. Typically, tissue fluids like milk, urine, serum, or gastric juice do not exhibit mutagenic activity unless exposed to external mutagens [[39], [40], [41]]. Ovulatory FF carries abundant inflammatory cytokines and ROS essential for tissue destruction and ovulation [42,43], which may contribute to its mutagenic activity.

To imitate the sequential exposure of FTE to FF after ovulation, we designed Early and Late exposure modes and found that both could equally induce mutagenesis in exposed cells. Interestingly, the transient 2-min exposure to 100 % FF exerted the same magnitude of mutagenesis as in the long-lasting exposure to 10 % FF. This suggests that the magnitude of mutagenic activity is not dependent on the dose or duration of FF exposure but rather on the extent of AID activation. We also found that non-transformed FE25 (P20) cells were approximately 2.5 and 4 times more vulnerable to mutagenesis than transformed FE25 (P90) and ovarian cancer-derived A2780 cells, respectively (Table 1). In the immunohistochemical (IHC) staining of AID in serous tubal intraepithelial carcinoma, Sapoznik et al. noted a significantly stronger AID signal in the adjacent normal epithelium than in the lesion [21], supporting our findings.

AID expression has been observed to increase during viral infections, triggering innate immune responses in various settings. For instance, hepatocytes respond to hepatitis B virus, [44] mouse primary B cells combat Abelson murine leukemia virus [45], and in human primary naïve B cells react to Kaposi's sarcoma-associated herpes virus [46] by upregulating AID. Moreover, studies on *Helicobacter pylori* infection of gastric epithelial cells [47] and inflammation of cecal mucosa in an inflammatory bowel disease mouse model [48] have reported dysregulated AID expressed. The current study echoes a previous finding that ovulatory FF can activate AID [21] and further elucidates the detailed time sequence of the AID response and consequent mutagenesis in accordance with physiological exposure after ovulation. Early mutagenic activity was preceded by immediate induction of AID mRNA and protein, starting at 0 min and peaking at 30 and 45 min, respectively. Late mutagenic activity followed an increase in AID mRNA at 6 h and AID protein at 7 h after sustained exposure to 10 % FF. The Early and Late activity was mediated by the classical signaling proteins ERK and NF-κB, respectively. The biological significance of these two waves of AID activation in the FTE following ovulation remains unclear. It is plausible that AID activation serves the purpose of DNA demethylation and tissue remodeling following ovulatory injury [21,49], with mutagenesis occurring as an unintended consequence.

We found that ROS, which are abundant in the FF, are the fundamental cause and secondary mediators of AID induction. Treatment with equivalent amounts of H_2_O_2_ induced the same magnitude of AID upregulation, whereas cotreatment with NAC diminished this effect. Although TNF-α and estradiol, which are also present in FF at high levels, can boost AID expression, neither the TNF-α blocking antibody nor the estrogen-receptor antagonist mitigated the AID upregulation caused by FF exposure in a previous study [21]. The present study reveals that both TNF-α and estradiol rely on the ROS signal for AID induction, underscoring the essential role of ROS in FF exposure-induced AID activation and mutagenesis and suggesting an antioxidant strategy for the prevention of ovulation-induced mutagenesis.

*TP53*, known as the key responder to oxidative stress and DNA damage, emerges as a central target of AID in various inflammatory diseases. Evidence suggests that *TP53* mutations are prevalent in fibroblast-like synoviocytes in rheumatoid arthritis, with AID-positive cells exhibiting a heightened frequency of somatic *TP53* mutations [50]. Furthermore, in a mouse model of inflammatory bowel disease characterized by frequent *Trp53* mutations in the cecum, the absence of AID resulted in a significant reduction in mutation incidence. The findings of the present study provide deeper insights into the causal relationship between ROS, AID, and p53 mutation within the context of ovulation.

Early epidemiological studies on the long-term effects of ovulation inhibition have supported the notion that ovulation can initiate ovarian cancer. Women who have used oral contraceptives, even for a short time, are protected from ovarian cancer for >30 years after cessation of usage [51]. This implies that ovulation carries cancer risk spanning several decades prior to the diagnosis of ovarian cancer, a timeframe long enough for FTE oncogenesis [8]. During this early phase of risk, ovulation can only contribute to the initiation of malignant transformation. Hence, exposure to FF that induces TP53 gene deamination can serve as a cancer initiation event in the progression of HGSC. The early pathological feature referred to as the “p53 signature” could originate from an initial *TP53* mutation in a stem or progenitor cell, followed by clonal expansion. Furthermore, our previous research has demonstrated that the abundance of IGF axis proteins in FF promotes stemness activation and clonal expansion of the p53/Rb-initiated FTE cells [13].

## Conclusion

In conclusion, our investigation has unveiled the mutagenic properties of ovulatory FF by upregulating AID and affecting the TP53 gene. We have identified two distinct waves of mutagenic activity within the fimbria epithelium exposed to FF, both dependent on ROS. This research has elucidated the previously unidentified connection between ovulation and TP53 mutation, a pivotal event in the initiation of HGSC, and has underscored the potential effectiveness of antioxidant intervention as a preventive strategy.

## Consent for publication

Not applicable.

## Data sharing statement

Data available upon request.

## Funding statement

This study was supported by grants from the Ministry of Science and Technology, Taiwan (MOST111-2314-B303-018), and the National Science and Technology Council, Taiwan, ROC (NSTC 112-2314-B-303-002).

## CRediT authorship contribution statement

**Kanchana Subramani:** Data curation, Formal analysis, Investigation, Methodology, Validation, Writing – original draft. **Hsuan-Shun Huang:** Formal analysis, Investigation, Validation, Visualization. **Pao-Chu Chen:** Formal analysis, Resources, Visualization. **Dah-Ching Ding:** Formal analysis, Resources, Visualization. **Tang-Yuan Chu:** Conceptualization, Formal analysis, Funding acquisition, Resources, Supervision, Validation, Visualization, Writing – review & editing.

## Declaration of competing interest

The authors declare that they have no known competing financial interests or personal relationships that could have appeared to influence the work reported in this paper.

## Appendix Supplementary materials

Image, image 1

Image, application 2

Image, application 3
