# Corrigendum to “Ovulation sources ROS to confer mutagenic activities on the TP53 gene in the fallopian tube epithelium” [Neoplasia 59 (2025 Dec 4) 101085]

**DOI:** 10.1016/j.neo.2025.101183

**Published:** 2025-05-29

**Authors:** Kanchana Subramani, Hsuan-Shun Huang, Pao-Chu Chen, Dah-Ching Ding, Tang-Yuan Chu

**Affiliations:** a Center for Prevention and Therapy of Gynecological Cancers, Department of Medical Research, Hualien Tzu Chi Hospital, Buddhist Tzu Chi Medical Foundation, Hualien, Taiwan, ROC; b Institute of Medical Sciences, Tzu Chi University, Hualien, Taiwan, ROC; c Department of Obstetrics & Gynecology, Hualien Tzu Chi Hospital, Buddhist Tzu Chi Medical Foundation, Hualien, Taiwan, ROC

The authors regret to inform that on the “Clinical specimen” section in Method, the sentence “Approval for the collection of clinical specimens was obtained from the Institutional Review Board of Tzu Chi General Hospital, Hualien, Taiwan (TCGH-IRB #93-025).” should **have “(TCGH-IRB #93-025)” changed to “(IRB111-038-A)”.**

The authors would like to apologise for any inconvenience caused.

