# Supplementary figures and images for: Ovulation sources ROS to confer mutagenic activities on the TP53 gene in the fallopian tube epithelium
Source: Neoplasia. 2024 Dec 4;59:101085. doi: 10.1016/j.neo.2024.101085 (PMC11664131; doi:10.1016/j.neo.2024.101085)

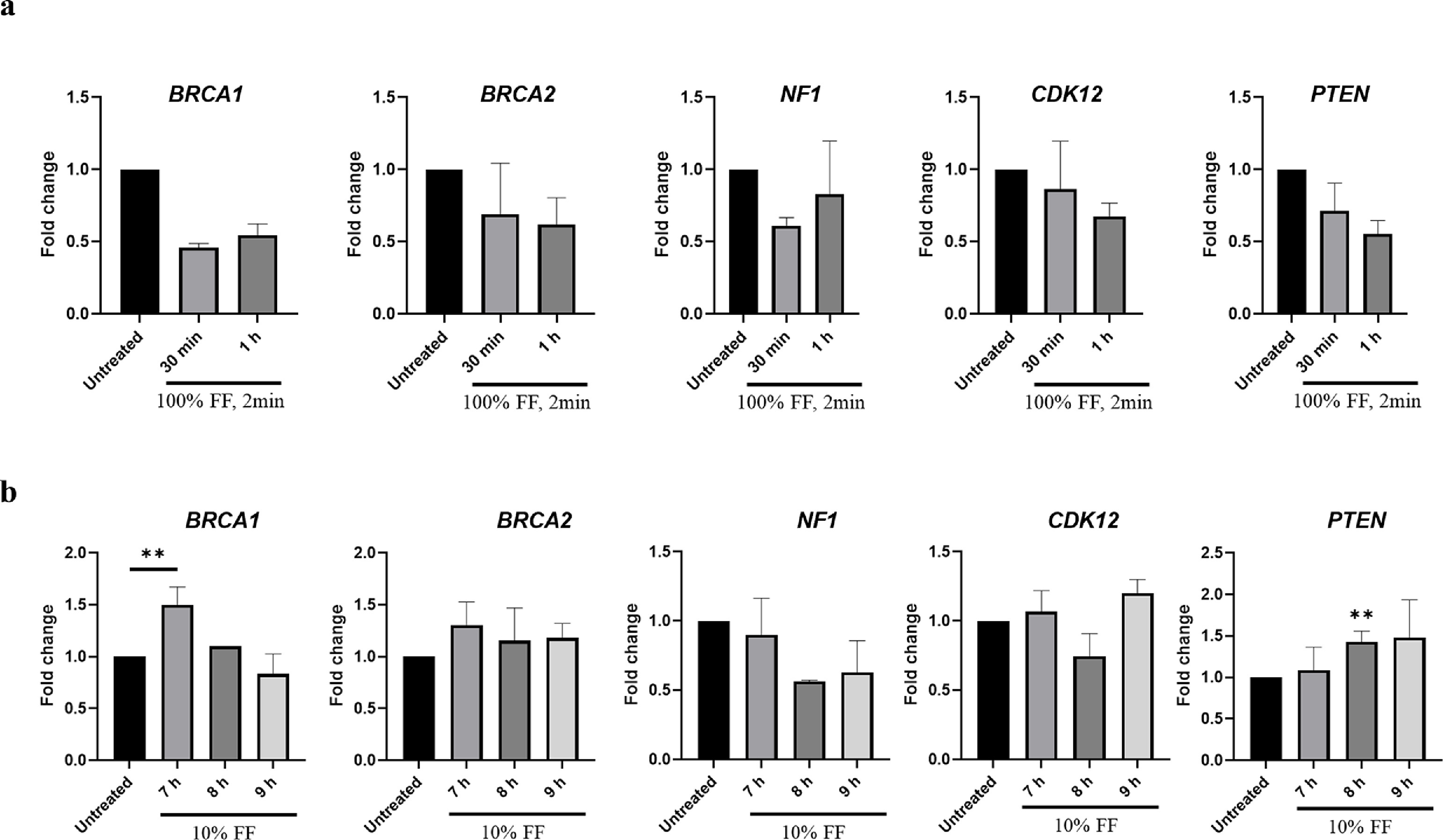

Supplement: Supplementary file 1 [file mmc1.jpg]
