# Supplementary material for: Ovulation sources ROS to confer mutagenic activities on the TP53 gene in the fallopian tube epithelium
Source: Neoplasia. 2024 Dec 4;59:101085. doi: 10.1016/j.neo.2024.101085 (PMC11664131; doi:10.1016/j.neo.2024.101085)
Supplement: Supplementary file 2 [file mmc2.docx]

**Suppl. Table 1: The plating efficiency of individual treatment groups in different cell lines**

| **Treatment groups** | **Concentration** | **Plating efficiency (%)** | | | | | |
| --- | --- | --- | --- | --- | --- | --- | --- |
|  |  | **CHO K1** | **A2780** | **FE25 (P20)** | **FE25**  **(P90)** | **FT282V** | **FT282**  **CCNE1** |
| **Control** |  | 98 | 100 | 98 | 100 | 96 | 100 |
| **EMS** | 500 µM | 100 | 100 | 92 | 100 | 97 | 100 |
| **FF** | 10% | 98 | 99 | 98 | 100 | 100 | 96 |
| **FF** | 100% | 100 | 98 | 89 | 100 | 97 | 98 |

**EMS (Ethyl methane sulfonate), FF (Follicular fluid)**
