# Supplementary material for: Ovulation sources ROS to confer mutagenic activities on the TP53 gene in the fallopian tube epithelium
Source: Neoplasia. 2024 Dec 4;59:101085. doi: 10.1016/j.neo.2024.101085 (PMC11664131; doi:10.1016/j.neo.2024.101085)
Supplement: Supplementary file 3 [file mmc3.docx]

**Suppl. Table 2: The primer pairs used for PCR and qRT-PCR**

| **No.** | **Name of the primer** | **Primer sequences** | **PCR product length (bp)** |
| --- | --- | --- | --- |
| **1** | **TP53 (Exon 7)** | **5’GAGGTTGGCTCTGACTGTACC**  **5’TCCGTCCCAGTAGATTACCAC** | **476** |
| **2** | **TP53 (Exon 1)** | **5’CACAGCTCTGGCTTGCAGA**  **5’AGCGATTTTCCCGAGCTGA** | **442** |
| **3** | **TP53 (Exon 2)** | **5’AGCTGTCTCAGACACTGGCA**  **5’GAGCAGAAAGTCAGTCCCATG** | **317** |
| **4** | **TP53 (Exon 5)** | **5’CTCTGTCTCCTTCCTCTTCC**  **5’GCAATCAGTGAGGAATCAGAGG** | **284** |
| **5** | **TP53 (Exon 6)** | **5’AGATAGCGATGGTGAGCAGC**  **5’ACTGACAACCACCCTTAACC** | **258** |
| **6** | **TP53 (Exon 11)** | **5’TCCCGTTGTCCCAGCCTT**  **5’TAACCCTTAACTGCAAGAACAT** | **476** |
| **7** | **PTEN** | **5’ACGACGGGAAGACAAGTTCA**  **5’AGGTTTCCTCTGGTCCTGGT** | **3038** |
| **8** | **BRCA 1** | **5’TCAGCAAACCTAAGAATGTGGGATA**  **5’GGGAGCACATTTTACAAATTTCCAA** | **509** |
| **9** | **BRCA 2** | **5’ATTGCATTCTTCTGTGAAAAGAAGC**  **5’AGCACATACATCTTGATTCTTTTCCA** | **556** |
| **10** | **NF 1** | **5’TGAGTTTTAGAGGCTGTTAATTTGCT**  **5’CCATATATAGGTTCATTTCAGGCCCTA** | **538** |
| **11** | **CDK 12** | **5’TGGACTTGCTCGGCTCTATAACTC**  **5’CCCAAGAATACATCCACAGCTCCA** | **148** |
